# Tyrosine kinase inhibitors as potential sensitizers of adoptive T cell therapy for hepatocellular carcinoma

**DOI:** 10.3389/fimmu.2023.1046771

**Published:** 2023-03-01

**Authors:** Linjun Liang, Xiaoyan Wang, Shuying Huang, Yanwei Chen, Peng Zhang, Liang Li, Yong Cui

**Affiliations:** ^1^ Shenzhen Institutes of Advanced Technology, Chinese Academy of Sciences, Shenzhen, China; ^2^ Department of Oncology, Shenzhen Qianhai Shekou Free Trade Zone Hospital, Shenzhen, Guangdong, China; ^3^ Department of Pulmonary Critical Care Medicine of Tangdu Hospital, Fourth Military Medical University, Xi’an, China; ^4^ School of Medicine, Southern University of Science and Technology, Shenzhen, Guangdong, China

**Keywords:** hepatocellular carcinoma, tyrosine kinase inhibitors, adoptive T cell therapy, immunotherapy, combination therapy

## Abstract

Hepatocellular carcinoma (HCC) is a high-incidence malignant tumor worldwide and lacks effective treatment options. Targeted drugs are the preferred recommendations for the systemic treatment of hepatocellular carcinoma. Immunotherapy is a breakthrough in the systemic treatment of malignant tumors, including HCC. However, either targeted therapy or immunotherapy alone is inefficient and has limited survival benefits on part of HCC patients. Investigations have proved that tyrosine kinase inhibitors (TKIs) have regulatory effects on the tumor microenvironment and immune response, which are potential sensitizers for immunotherapy. Herein, a combination therapy using TKIs and immunotherapy has been explored and demonstrated to improve the effectiveness of treatment. As an effective immunotherapy, adoptive T cell therapy in solid tumors is required to improve tumor infiltration and killing activity which can be possibly achieved by combination with TKIs.

## 1 Introduction

According to GLOBOCAN2020 (a global cancer statistic online database), primary liver cancer, a common malignant tumor with high morbidity and mortality, ranks 6th in tumor incidence and 3rd in tumor-related mortality globally. Hepatocellular carcinoma (HCC) is the most common type of primary liver cancer (accounts for 75%-85%) (1). As the greatest etiological risk factor for HCC, viral infections including hepatitis B virus (HBV), hepatitis C virus (HCV), and hepatitis delta virus (HDV) are considered the major cause of HCC cases (2). Liver fibrosis and cirrhosis, caused by chronic hepatitis infections, will further affect the patients’ liver function and the anti-tumor therapy, reducing the survival rate of patients. The high mortality rate of HCC is attributed to its insidious nature and lack of effective treatment options. Early-stage HCC patients with hepatic resection and transplantation have a 5-year survival of 70–80% (3). However, the majority of HCC patients are diagnosed in the intermediate stages or advanced stages resulting in a significant decrease in survival rate due to missing the best treatment opportunities and lack of effective therapies (4, 5).

Tyrosine kinase inhibitors (TKIs) with antiangiogenic properties are used as first-line systemic therapy in advanced HCC. Although TKIs have certain survival benefits for HCC patients, the inevitable problem of primary or acquired resistance has become the bottleneck that interferes the long-term response and limits the improvement of prognosis. In addition to resistance issues, the lack of identified driver mutations, underlying liver cirrhosis, and impaired functional reserves are also difficulties in treating advanced HCC patients.

Adoptive T cell therapy is an emerging research topic in tumor immunotherapy that progress rapidly from basic research to clinical application. Adoptive T cell therapy refers to manipulating and expanding autologous or allogeneic T cells *in vitro* followed by infusing into tumor patients to generate a robust immune-mediated antitumor response (6). Previous research has demonstrated the promising efficacy of adoptive T cell therapy in the treatment of hematologic tumors. There are growing investigations conducted to evaluate adoptive T cell therapy in solid tumors, including HCC. However, several barriers are identified, including the immunosuppression of the tumor microenvironment (TME) (7, 8) and target antigen heterogeneity (9). To improve the survival time and living quality of HCC patients, researchers are engaged in overcoming these issues mentioned above.

Recently, it has been discovered that TKIs also have immunomodulatory effects besides their well-known antiangiogenic properties (10). This finding obtained great attention to the combination therapy of TKIs with other immune therapies. In this review, we discuss this new combination therapy with a focus on TKIs as potential sensitizers of adoptive T cells for HCC. In the beginning, an introduction to diagnostic methods and current treatment strategies will be given. The discovery of immunomodulatory effects of TKIs and the underlying mechanisms will then be presented. Finally, current adoptive T cell therapies for HCC together with their combination therapies with different TKIs will be discussed.

## 2 Diagnoses and treatments for HCC

### 2.1 Diagnoses for HCC

Diagnostic imaging and Alpha-fetoprotein (AFP) testing are the most common ways for HCC screening and diagnosis (3). However, imaging methods, including ultrasound, CT, and MRI, are less effective for HCC diagnosis at early stage (11, 12). As to AFP testing, a serum biomarker for HCC normally used in conjunction with imaging, it is not sensitive or specific for HCC that about 30% of HCC patients with confirmed liver cancer have no significant increase in AFP (13). Since more curative approaches are available to early-stage HCC patients resulting in a higher survival rate, advanced screening techniques and diagnostic methods capable of detecting HCC at early stage are required to gain the optimal therapeutic outcome (3).

Recently, new screening techniques have been studied and shown advantages in early screening and early diagnosis of liver cancer such as the liquid biopsy method which is based on circulating tumor cells (CTC), circulating tumor DNA (ctDNA), extracellular vehicles (EVs), circulating cell-free RNA (cfRNA) (14, 15). It has been reported that ctDNA is superior to serum AFP in sensitivity and specificity for early screening of HCC (16). In addition, the development of molecular biology technology has discovered the important role of non-coding RNA (ncRNA) in tumorigenesis and progression, providing alternative ways for early-stage HCC detection. ncRNAs, taking up to 60% of the transcriptional output in human cells, are untranslated transcripts and can be classified based on their length, such as microRNAs (22-25 nucleotides) and long-ncRNAs (>200 nucleotides) (17, 18). Researchers have found abnormal expression of miRNA in liver cancer, such as miRNA-502c-3p, miRNA-342-3p, and miRNA-21 (19–22). The accuracy of using a miRNA panel (consisting of 7 miRNAs) can improve the diagnosis of HCC to reach a detection rate close to 90% (23). LncRNA is also associated with cancer progression and can function as HCC diagnostic markers such as TUG1, ZFAS1, and SCARNA10 (21, 24–26). Compared with traditional tumor markers, ncRNA expression abnormalities may appear earlier and can potentially become a category of more convenient, accurate, and non-invasive detection markers.

### 2.2 Current treatments for HCC

Tumors can be characterized by The Barcelona Clinic Liver Cancer (BCLC) staging system *via* performance status, the size and number of tumors, vascular invasion, extrahepatic metastases, and liver function (27). Treatment strategies for tumors in each stage are varied. Liver transplantation, hepatic radical resection, and ablation are preferred and applicable to early-stage HCC. Surgery, including hepatic resection and liver transplantation, has been considered as curative therapy, which is currently the most important way to help patients achieve long-term survival (28–31). Hepatic resection is recommended for patients with excellent performance status, good liver function, and no clinically significant portal hypertension (32). Ablation delivers heat directly to tumors to induce tumor necrosis. It is also a potentially curative procedure for HCC at early stages, with similar survival rates to surgical resection (33–35). As to intermediate-stage HCC patients, transarterial chemoembolization (TACE) is the first candidate. The current treatments for advanced HCC are systemic therapies, including chemotherapy, targeted therapy, and immunotherapy (36–38). Though curative and locoregional therapies showed promising prognoses, more than 60% of HCC patients were diagnosed at the middle and advanced stages, eventually receiving systemic therapy. Systemic therapy thus plays a vital role in liver cancer treatment.

Since chemotherapy showed unsatisfactory outcomes and related to severe toxicities, molecularly targeted therapy and immunotherapy have played a critical role in the systemic treatment of advanced HCC (39–41). Several molecular targeted therapy drugs with effects on angiogenesis, cell proliferation, and metastasis have been shown to improve overall survival (OS) in advanced HCC. The main molecular targeted drugs in use are TKIs and monoclonal antibodies.

TKIs belong to a category of tumor angiogenesis inhibitors. Sorafenib is the first multikinase inhibitor approved for the treatment of HCC based on its benefit in two multicenter clinical trials (42–44). Sorafenib works through the inhibition of the receptor tyrosine kinases of vascular endothelial growth factor receptor (VEGFR), platelet-derived growth factor receptor (PDGFR), fibroblast growth factor receptor(FGFR)-β, and c-KIT, blocking the RAF/MEK/ERK pathway (45, 46). Lenvatinib showed a non-inferiority in overall survival and superiority in progression-free survival, time to progression and objective response rate (ORR) compared to sorafenib in a randomized phase 3 non-inferiority trial and has been approved as another first-line treatment of HCC (47). The second-line TKIs are cabozantinib and regrafinib. These agents commonly have effects targeting various receptors including VEGFR, PDGFR, and FGFR (48).

For monoclonal antibodies, bevacizumab and ramucirumab are currently used in clinics based on the promising results obtained from previous clinical studies. The combination of bevacizumab (a monoclonal antibody that targets VEGF, inhibiting angiogenesis) and atezolizumab (an immune checkpoint inhibitor that targets programmed cell death protein-1 and reverses T-cell suppression) has become one of the first-line treatment because it showed better overall and improved survival with unresectable HCC compared with sorafenib in IMbrave150, a global phase III randomized trial (49). Ramucirumab is a human immunoglobulin G1 monoclonal antibody and a VEGFR2 inhibitor. It is now a second-line therapy because, in the REACH-2 trial, ramucirumab showed a significant improvement compared with placebo in median OS(mOS) for advanced HCC patients who had prior sorafenib treatment (50).

In the last two decades, significant breakthroughs have been made in immunotherapy for tumors, including monoclonal antibodies, tumor vaccines, adoptive transfer of immune cells, and immunomodulatory agents (39, 51). Immune checkpoints are key members of immunoregulatory pathways regulated by ligand/receptor interactions, playing an important role oftenly in preventing overactive immune responses. Immune checkpoint inhibitors (ICIs) are monoclonal antibodies that regulate T cell activity through a series of targets, such as programmed cell death protein-1 and its ligand (PD-1/PD-L1), cytotoxic T lymphocyte protein 4 (CTLA-4), mucin domain-containing molecule-3 (Tim-3), and lymphocyte activation gene 3 protein (LAG-3), to exert anti-tumor effects (40). Including atezolizumab, pembrolizumab, ipilimumab, and nivolumab, ICIs and their combination regimens have been used as first-line and second-line treatments for hepatocellular carcinoma in clinical practices (52). In addition to the above commonly used treatment strategies, antiviral therapy is also an optional method considering that chronic HBV/HCV infection is a major cause of HCC. Studies have shown antiviral therapy benefits the survival of HBV-associated HCC patients (53, 54). For example, anti-HBV drugs have been reported to suppress the growth of HBV-expressing hepatoma cells *via* down-regulation of the hepatitis B virus X protein (55).

Although the HCC treatment options and their efficiency have been developed in recent years, and the application of multidisciplinary treatment strategies shows better outcomes compared with monotherapies, the current objective remission rate and the prognosis are still unsatisfactory (56). The obstacles, such as the recurrence rate of surgery, inevitable drug resistance of targeted drugs, and a low response to systemic cancer therapy, are at urgent need to be overcome.

## 3 The discovery and underlying immunomodulatory mechanisms of TKIs

Recently, new treatments and optimization of current treatment regimens through combination therapies have been studied to improve prognosis. Several clinical trials have been conducted and showed promising outcomes. For example, several new molecular kinase inhibitors like palbociclib, ribociclib, and tivantinib have been under investigation and have shown their effectiveness (57, 58). As to combination therapies, an interesting finding has been reported that TKIs possess synergistic antitumor effects with immunotherapies in the clinical trials of combination therapy (58). In this section, we will introduce the discovery of TKIs’ immunomodulatory effect first and then discuss the related mechanisms.

### 3.1 The discovery of immunomodulatory effects of TKIs

The immunomodulatory effect of TKIs was discovered in combination therapies. Both preclinical and clinical trials of combinations of anti-PD-1 and TKIs have shown promising outcomes for HCC treatments (59). In a phase Ib open-label multicentre study, 100 HCC patients were enrolled, who were diagnosed as BCLC stage B (n=29) or C (n=71) diseases. The result showed that the ORR of lenvatinib plus pembrolizumab treatment was 46.0% (95% CI, 36.0% to 56.3%) by modified Response Evaluation Criteria in Solid Tumors (mRECIST), without new or unexpected toxicities resulting from the combination therapy (60). Notably, 11 patients (11%) were evaluated to have a complete response (CR). At the data cut-off date, median progression-free survival (mPFS) was 9.3 months (95% CI, 5.6 to 9.7 months) and mOS was 22.0 months (95% CI, 20.4 months to NE), showing a promising antitumor activity observed in unresectable HCC with lenvatinib plus pembrolizumab treatment. Immunomodulatory effects of TKIs were also observed in mouse models of liver cancer. Kenichi Nomoto et al. found that lenvatinib had greater antitumor activity in immunocompetent mice than in immunodeficient mice, which implies that part of the antitumor effect of lenvatinib originates from the immune responses (61). It was further demonstrated that lenvatinib plus anti-PD-1 antibody enhanced tumor regression and increased treatment response rate. Both studies showed an increased percentage of CD8^+^ T cells, confirming the synergistic effect of TKIs and immunotherapy (61, 62).

### 3.2 The underlying immunomodulatory mechanism of TKIs

**Figure 1:**
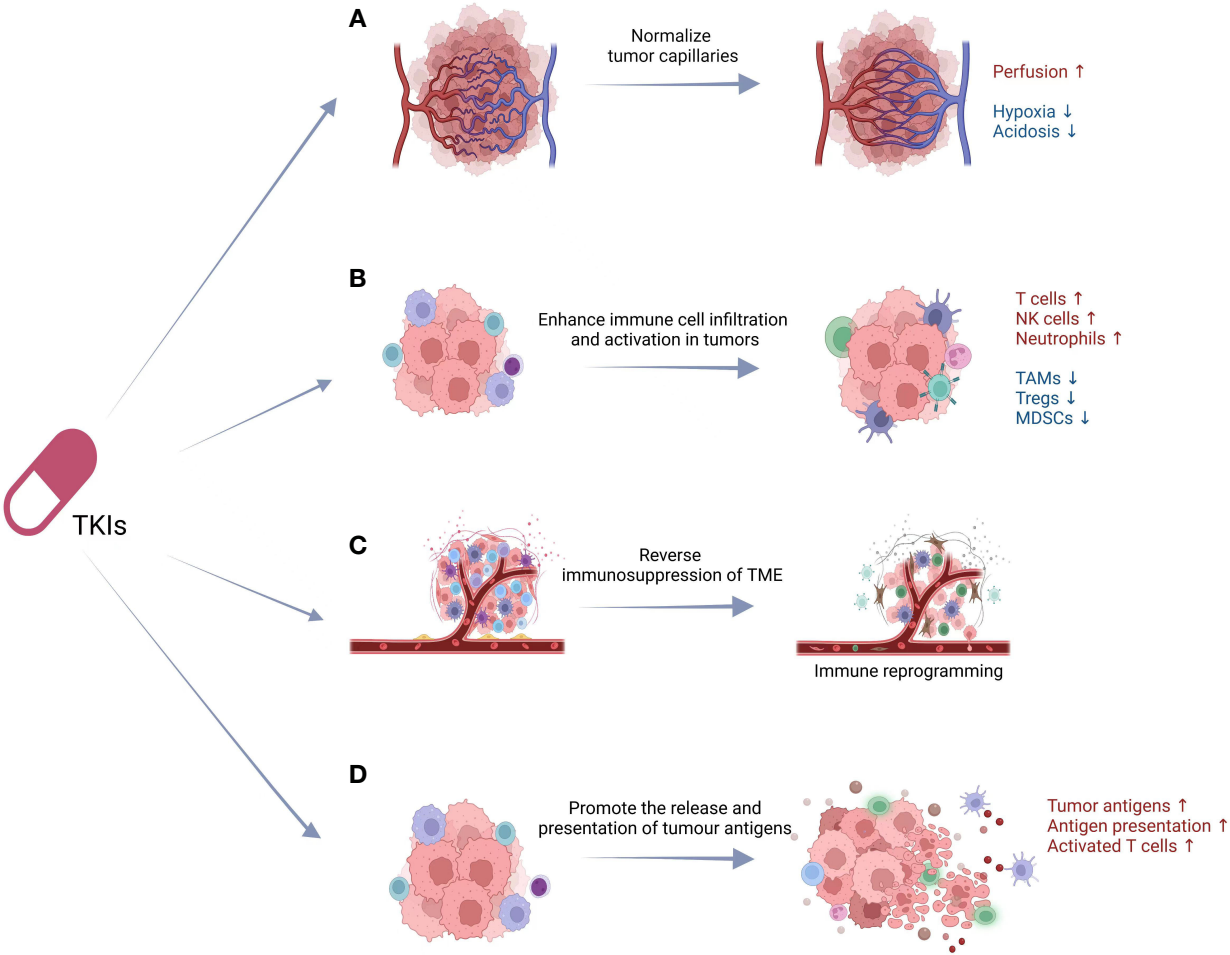
The underlying immunomodulatory mechanisms of TKIs. The immunomodulatory effects of TKIs are based on the following: **(A)** normalize tumor capillaries, **(B)** enhance immune cell infiltration and activation in tumors, **(C)** reverse immunosuppression of TME, **(D)** promote the release and presentation of tumor antigens. TKIs, tyrosine kinase inhibitors; TAMs, tumor-associated macrophages; Tregs, regulatory T cells; MDSCs, myeloid-derived suppressor cells; TME, tumor microenvironment; DCs, dendritic cells.

Targeting on the varied functional mechanisms of the therapeutic targets, TKIs induce immunomodulatory effects with increased antitumor effects and decreased immunosuppression based on the following main categories of mechanisms: (a) normalize tumor capillaries, (b) enhance immune cell infiltration and activation in tumors, (c) reverse immunosuppression of TME, (d) promote the release and presentation of tumor antigens (**Figure 1**).

Different from normal vessels, pathological angiogenesis in tumors shows abnormal structures and functional defects (63). Pathologic neovascularization is the critical manner of tumor blood supply, which further induced hypoperfusion, hypoxia, acidosis, and tumor metastasis (64, 65). Neovascularization is regulated by more than 40 molecules acting proangiogenicly or antiangiogenicly, respectively (66–68). Angiogenesis inhibitors including TKIs are first applied to decrease tumor vessel formation, resulting in blocking nutrient supply and inducing tumor cell dormancy (63). Unexpectedly, the alleviation of hypoxia and acidosis in tumor tissue has been found in clinical application when using lower doses of antiangiogenic agents (69), which may synergize with radiotherapy, chemotherapy, and immune therapy. Rakesh K. Jain first proposed the rationale that antiangiogenic therapy normalizes tumor vasculature networks before their destruction and hence improves the delivery of oxygen (70). This rationale based on the antiangiogenic therapy rectifies the imbalance of proangiogenic and antiangiogenic factors, which results in the elimination of the excess endothelial cells and the immature and inefficient blood vessels. Subsequent pre-clinical studies verified this hypothesis and revealed that inhibition of multiple tumor-promoting effects of VEGF is the key factor (63). However, sustained antiangiogenic therapy cannot maintain its effect in normalizing the pathogenic tumor vessels. Instead, sustained antiangiogenic results in the reduction rather than normalization of pathogenic tumor vessels. Thus, extensive experiments are still needed to explore an optimized strategy of vascular normalization which is potentially applicable in clinical practice.

Besides the effect of normalizing tumor vasculature, TKIs can also improve the infiltration and activation of antitumor immune cells to interfere with tumor progression. CD8^+^ T cells are the most important type of tumor-killing cells, and TKIs significantly increase the proportion of anti-tumor effector T cells (61, 71). Another investigation showed that TKIs enhanced the effector functions of human NK cells by activation of the RAS/RAF/ERK pathway in a dose- and time-dependent manner (72). TKIs have been reported to induce increased expression of recognizing and activating ligands for NK cells, T cells, NKT cells, NKT-like cells, and γσT cells, promoting chemotaxis and tumor killing (73–76). Down-regulation of major histocompatibility complex class I (MHC-I) expression of tumor cells by TKIs is also a major underlying mechanism to increase NK cell responses (77). Moreover, TKIs can induce tumor cells to release neutrophil chemotactic factors, resulting in a rapid neutrophil infiltration into the tumor and enhanced tumor eradication (78).

In addition to insufficient immune infiltration, immunosuppressive factors in TME also prevent immune cells from exerting anti-tumor effects. TKIs can reverse the immunosuppression of TME by reducing the infiltration and immunosuppressing effect of tumor-associated macrophages (TAMs), regulatory T cells (Tregs), and myeloid-derived suppressor cells (MDSCs) (79). TAM is involved in the formation of the immunosuppressive microenvironment and the inhibition of anticancer immune responses by secreting immunosuppressive factors, inhibiting the proliferation and activation of T cells, and promoting tumor invasion and metastasis (80). Ulrike Protzer et al. investigated the influence of sorafenib on TAM *in vitro* and *in vivo*. The results revealed that sorafenib stimulated macrophages to produce cytokines such as IL-6, TNF-a, and IL-12, and subsequently induced antitumor NK cell responses *via* the NF-κB pathway (81). Other studies validated sorafenib’s modulatory effect on TAM by decreasing the percentage of TAMs and restoring the classical activation of macrophages, thus decreasing the tumor burden (77, 81, 82).

Interactions of TKIs and cancer cells are considered to mediate immunomodulatory activity by promoting tumor antigen presentation. T cells are known to specifically recognize tumour antigens and become activated to execute their anti-tumor functions, while insufficient presentation of tumour antigens is an important reason for the lack of T cell responsiveness in tumor patients (83). TKIs induce cytotoxicity through the inhibition of multiple tyrosine kinases to block the VEGFR and PDGFR pathways. Subsequent cell death can expose tumour antigens and activate antigen presentation by dendritic cells (DCs). This cross-presentation of antigens activates T cells, completing a so-called “immune sensitization” (83, 84). On the other hand, VEGF inhibits the adhesion of lymphocytes to endothelial cells through intercellular adhesion molecule-1 (ICAM-1), vascular cell adhesion molecule-1 (VCAM-1), and Fas Ligand (FasL), thereby affecting the infiltration of T cells into the tumor tissue (85, 86). In addition, VEGF inhibits the maturation of DCs, and the development and activation of T cells (87, 88). Negative regulation of VEGF functions on DCs and T cells is the potential mechanism of TKIs promoting antigen presentation.

## 4 TKIs as potential sensitizers of adoptive T cell therapy for HCC

Showing a promising antitumor activity, the landscape of treatment strategy for HCC may change dramatically with the advance of immunotherapy in the near future. Since the immunomodulation effect of TKIs has been discovered, combination therapy of TKIs with other immunotherapies has also been under evaluation, such as ICIs and immune cells (89). Adoptive T cell therapy is one of the immunotherapies and has shown great value for clinical translation. Currently, several adoptive T cell therapies have been approved by Food and Drug Administration (FDA) for lymphoma, myeloma, and leukemia (90). In recent years, there are many attempts in applying adoptive T cell therapies for other tumors. In this section, we will first summarize the current state of applying adoptive T cell therapy for HCC and discuss the related challenges. The combination therapy of TKIs with adoptive T cells will also be discussed.

### 4.1 Adoptive T cell therapy for HCC

T cells are important effector cells in the specific immune response of the somatic machinery to tumor cells and are currently the most widely studied effector cells in pericyte therapy. T cell-based pericyte therapies include autologous T cells, allogeneic T cells, tumor-infiltrating lymphocytes (TILs), chimeric antigen receptor T cells (CAR-T cells), T cell receptor engineered T cells (TCR-T cells) and other engineered T cells (9). Up to date, 63 researches of T cell-based therapies for HCC have been registered at ClinicalTrials (https://clinicaltrials.gov/ ), with CAR-T cells account for the largest proportion among all T cell transfer approaches (**Table 1**). CAR-T cells originally showed their remarkable efficacy in treating hematological malignancies (91). The chimeric antigen receptors (CARs) contain an extracellular antigen-recognition domain, a transmembrane domain, and an intracellular signaling domain, which recognize tumor-specific antigens expressed and achieve non-MHC-restricted activations (92). Glypian-3 (GPC-3) has been demonstrated to relate with immunoreactivity towards tumor cells and is highly expressed in HCC, becoming a notable candidate target in HCC. CAR-T cells targeting GPC-3 have been shown effective in animal models with orthotopic xenografts and patient-derived xenografts highly expressing GPC-3 (93, 94).

**Table 1:** Clinical Trials of Adoptive T Cell Therapy for HCC Registered at ClinicalTrials from 2008 to 2022.

NCT Number	Type of T cells	Title	Phase
NCT03175705	Autologous T cells	Adoptive Transfer of Specific HCC Antigens CD8+ T Cells for Treating Patients With Relapsed/Advanced HCC	I
NCT05304481	Autologous T cells	Efficacy and Safety of Activated T Lymphocytes (ATL) in Hepatocellular Carcinoma	II
NCT00562666	Autologous T cells	Immunotherapy of Hepatocellular Carcinoma With Gamma Delta T Cells	I
NCT03093688	Autologous T cells	Clinical Safty and Efficacy Study of Infusion of iNKT Cells and CD8+T Cells in Patients With Advanced Solid Tumor	I/II
NCT04518774	Allogeneic T cells	Allogeneic “Gammadelta T Cells (γδ T Cells)” Cell Immunotherapy in I Hepatocellular Carcinoma Clinical Trial	I
NCT02905188	CAR-T cells	Glypican 3-specific Chimeric Antigen Receptor Expressing T Cells for Hepatocellular Carcinoma (GLYCAR)	I
NCT05103631	CAR-T cells	Interleukin-15 Armored Glypican 3-specific Chimeric Antigen Receptor Expressed in Autologous T Cells for Hepatocellular Carcinoma	I
NCT04506983	CAR-T cells	GPC3-CAR-T Cells for the Hepatocellular Carcinoma	I
NCT03146234	CAR-T cells	CAR-GPC3 T Cells in Patients With Refractory Hepatocellular Carcinoma	Not Applicable
NCT05352542	CAR-T cells	GPC3-targeting LCAR-H93T Cell in Treatment of Advanced Hepatocellular Carcinoma	I
NCT03884751	CAR-T cells	Chimeric Antigen Receptor T Cells Targeting Glypican-3	I
NCT05003895	CAR-T cells	GPC3 Targeted CAR-T Cell Therapy in Advanced GPC3 Expressing Hepatocellular Carcinoma (HCC)	I
NCT04121273	CAR-T Cells	GPC3-targeted CAR-T Cell for Treating GPC3 Positive Advanced HCC	I
NCT03980288	CAR-T cells	4th Generation Chimeric Antigen Receptor T Cells Targeting Glypican-3	I
NCT03672305	CAR-T cells	Clinical Study on the Efficacy and Safety of c-Met/PD-L1 CAR-T Cell Injection in the Treatment of HCC	I
NCT05323201	CAR-T cells	Study Of B7H3 CAR-T Cells in Treating Advanced Liver Cancer	I/II
NCT02723942	CAR-T cells	CAR-T Cell Immunotherapy for HCC Targeting GPC3	I/II
NCT03084380	CAR-T cells	Anti-GPC3 CAR-T for Treating GPC3-positive Advanced Hepatocellular Carcinoma (HCC)	I/II
NCT05155189	CAR-T cells	A Study to Evaluate Safety and Efficacy of Armored CAR-T Cell Injection C-CAR031 in Advanced Hepatocellular Carcinoma	I
NCT03349255	CAR-T cells	Clinical Study of ET1402L1-CAR T Cells in AFP Expressing Hepatocellular Carcinoma	I
NCT04093648	CAR-T cells	T Cells co- Expressing a Second Generation Glypican 3-specific Chimeric Antigen Receptor With Cytokines Interleukin-21 and 15 as Immunotherapy for Patients With Liver Cancer (TEGAR)	I
NCT04951141	CAR-T cells	Clinical Study of Intratumoral Injection of CAR-T Cells in the Treatment of Advanced Liver Tumors	I
NCT02395250	CAR-T cells	Anti-GPC3 CAR T for Treating Patients With Advanced HCC	I
NCT05344664	CAR-T cells	Novel GPC3 CAR-T Cell Therapy for Hepatocellular Carcinoma	I
NCT03993743	CAR-T cells	A Study of CD147-targeted CAR-T by Hepatic Artery Infusions for Very Advanced Hepatocellular Carcinoma	I
NCT03198546	CAR-T cells	GPC3-CAR-T Cells for Immunotherapy of Cancer With GPC3 Expression	I
NCT02715362	CAR-T cells	A Study of GPC3 Redirected Autologous T Cells for Advanced HCC	I/II
NCT04270461	CAR-T cells	NKG2D-based CAR T-cells Immunotherapy for Patient With r/r NKG2DL+ Solid Tumors	I
NCT02587689	CAR-T cells	Phase I/II Study of Anti-Mucin1 (MUC1) CAR T Cells for Patients With MUC1+ Advanced Refractory Solid Tumor	I/II
NCT05131763	CAR-T cells	NKG2D-based CAR T-cells Immunotherapy for Patient With r/r NKG2DL+ Solid Tumors	I
NCT03130712	CAR-T cells	A Study of GPC3-targeted T Cells by Intratumor Injection for Advanced HCC (GPC3-CART)	I/II
NCT04550663	CAR-T cells	NKG2D CAR-T(KD-025) in the Treatment of Relapsed or Refractory NKG2DL+ Tumors	I
NCT05120271	CAR-T cells	BOXR1030 T Cells in Subjects With Advanced GPC3-Positive Solid Tumors	I/II
NCT05028933	CAR-T cells	IMC001 for Clinical Research on Advanced Digestive System Malignancies	I
NCT03013712	CAR-T cells	A Clinical Research of CAR T Cells Targeting EpCAM Positive Cancer	I/II
NCT03302403	CAR-T cells	Clinical Study of Redirected Autologous T Cells With a Chimeric Antigen Receptor in Patients With Malignant Tumors	Not Applicable
NCT03965546	CAR-T cells	ET 140202 -T Cell Combined With TAE or Sorafenib in the Treatment of Liver Cancer	I
NCT03888859	CAR-T cells	ET1402L1-ARTEMIS2 T Cells in Alpha Fetoprotein (AFP) Expressing Hepatocellular Carcinoma	I
NCT03941626	CAR-T cells/TCR-T cells	Autologous CAR-T/TCR-T Cell Immunotherapy for Solid Malignancies	I/II
NCT03638206	CAR-T cells/TCR-T cells	Autologous CAR-T/TCR-T Cell Immunotherapy for Malignancies	I/II
NCT04677088	TCR-T cells	TCR-Redirected T Cell Treatment in Patients With Recurrent HBV-related Hepatocellular Carcinoma Post Liver Transplantation	I
NCT04745403	TCR-T cells	Redirected HBV-Specific T Cells in Patients With HBV-related HCC (SAFE-T-HBV)	I
NCT02686372	TCR-T Cells	A Study of TCR-Redirected T Cell Infusion to Prevent Hepatocellular Carcinoma Recurrence Post Liver Transplantation	I
NCT03971747	TCR-T cells	AFP Specific T Cell Receptor Transduced T Cells Injection(C-TCR055) in Unresectable Hepatocellular Carcinoma	I
NCT04502082	TCR-T cells	Study of ET140203 T Cells in Adults With Advanced Hepatocellular Carcinoma (ARYA-1)	I/II
NCT03998033	TCR-T cells	Study of ET140202 T Cells in Adults With Advanced Hepatocellular Carcinoma	I
NCT04634357	TCR-T cells	ET140203 T Cells in Pediatric Subjects With Hepatoblastoma, HCN-NOS, or Hepatocellular Carcinoma	I/II
NCT02719782	TCR-T cells	A Study of TCR-Redirected T Cell Infusion in Subject With Recurrent HBV-related HCC Post Liver Transplantation	I
NCT05195294	TCR-T cells	Study of HBV-TCR T Cells (LioCyx-M) as Monotherapy or as Combination With Lenvatinib for HBV-related HCC	I/II
NCT03899415	TCR-T cells	TCR-Redirected T Cells Therapy in Patient With HBV Related HCC	I
NCT04368182	TCR-T cells	AFP Specific T Cell Receptor Transduced T Cells Injection(C-TCR055) in Unresectable Hepatocellular Carcinoma	I
NCT04756648	TCR-T cells	Phase I Clinical Trial of CT0180 Cells in the Treatment of Hepatocellular Carcinoma	I
NCT04973098	TCR-T cells	Phase I Clinical Trial of CT0181 Cells in the Treatment of Hepatocellular Carcinoma	I
NCT04864054	TCR-T cells	ECT204 T-Cell Therapy in Adults With Advanced HCC	I/II
NCT01967823	TCR-T cells	T Cell Receptor Immunotherapy Targeting NY-ESO-1 for Patients With NY-ESO-1 Expressing Cancer	II
NCT05339321	TCR-T cells	Autologous HBV-specific T Cell Receptor Engineered T Cells (TCR-T) in Patients With HBV-related Advanced HCC	I
NCT03132792	TCR-T cells	AFP-c332 T Cell in Advanced HCC	I
NCT02638857	Dendritic cell precision multiple antigen T cells	Immunotherapy Using Precision T Cells Specific to Multiple Common Tumor-Associated Antigen Combined With Transcatheter Arterial Chemoembolization for the Treatment of Advanced Hepatocellular Carcinoma	I/II
NCT02632188	Dendritic cell precision multiple antigen T cells	Radical Surgery Followed by Immunotherapy Using Precision T Cells Specific to Multiple Common Tumor-Associated Antigen for the Treatment of Hepatocellular Carcinoma	I/II
NCT04417764	PD-1 knockout engineered T cells	TACE Combined With PD-1 Knockout Engineered T Cell in Advanced Hepatocellular Carcinoma.	I
NCT03983967	Cytokine induced killer cells	Evaluate the Efficacy and Safety of ‘Immuncell-LC’ in Patients Undergoing Liver Transplantation	I/II
NCT02856815	Cytokine induced killer cells	Safety and Efficacy of “Immuncell-LC” in TACE Therapy	II
NCT01462903	Tumor infiltrating lymphocytes	A Study of Adoptive Immunotherapy With Autologous Tumor Infiltrating Lymphocytes in Solid Tumors	I

Adoptive T cell therapy can specifically and efficiently kill target cells. However, there are barriers and challenges for the clinical application of adoptive T cells. Theoretically, adoptive T cells can enhance cellular immune responses to eliminate cancer cells, but adoptive T cell therapy has demonstrated low clinical efficacy in the treatment of solid tumors, including HCC, due to the limited expansion, poor persistence, terminal differentiation and dysfunction or exhaustion of T cells (95, 96). Target antigen heterogeneity is also a barrier for adoptive T cell therapy application in solid tumors (9). Contrary to hematologic malignancies, solid tumors usually lack consistently expressed specific antigens for T cells to identify and become activated, consequently causing adoptive T cells fail to exert their anti-tumor effects. Off-target effects and cytokine storm also hinder the clinical performance of adoptive T cell therapy. In addition, the immunosuppression effect of the TME is a major barrier for the recruitment of adoptive T cells into tumors, fulfilling their antitumor activity (7, 8). TME is a complex circumstance including blood vessels, immune cells, fibroblasts, various signaling molecules, and extracellular matrix (97). Accumulation of immune cells, such as TAMs, MDSCs, and Tregs, as well as cytokines, including transforming growth factor-β, interleukin-6, and interleukin-1, are known to induce immunosuppression. As a result of interactions between cytokines, immune cells, and tumor cells, TME participates in the regulation of metastasis and anticancer drug sensitivity (97). In HCC, the immune-suppressing liver environments and chronic inflammation caused by liver diseases interfere with the effects of immunotherapy (98). There is an urgent need to more comprehensively understand adoptive T cell therapy effects and mechanisms, and optimize its application strategies.

### 4.2 TKIs as potential sensitizers of adoptive T cell therapy for HCC

As discussed in section 3, TKIs have been found having immunomodulatory effects and have shown improved patient outcomes by combination therapies. Together with the obstacles faced by current adoptive T cell therapy for HCC, it is rational to consider the possibility of combining TKIs with adoptive T cell therapy to achieve better performance. We here summarize the immunomodulatory effects of commonly used TKIs and discuss their potential applications as sensitizers in combination therapies with adoptive T cells.

The immunoregulatory effects of sorafenib have been observed recently. Sorafenib may enhance the infiltration and activation of T cells in the tumor microenvironment for various types of tumors by increasing the production of T cell-recruiting cytokines and chemokines (99–101). Treatment of serial low doses of sorafenib enhanced the activation, cytotoxicity, and migration of CD8^+^ T cells (102). Sorafenib inhibits the expression of multiple immunosuppressive factors, such as indoleamine 2,3-dioxygenase(IDO), transforming growth factor-beta (TGF-beta), VEGF, interleukin-10 (IL-10), monocyte chemoattractant protein-1 (MCP-1), by down-regulation of the STAT3 signaling pathway (102). In HCC, several studies have proven sorafenib can enhance antitumor responses through increasing the proportion of tumor-specific effector CD8^+^ T cells and reducing the proportion of exhausted or immunosuppressive immune cells such as PD-1-expressing CD8^+^ T cells and Foxp3+ Tregs (103, 104). The enhancement of antitumor immunity of sorafenib implies its potential as a sensitizer for adoptive T cell therapy. A recent study has confirmed this theory that combined treatment of GPC3-specific CAR-T cell therapy with subpharmacologic doses of sorafenib demonstrated an enhanced antitumor effect both *in vitro* and *in vivo* (105). Lenvatinib showed better antitumor activity than that of sorafenib in immunocompetent mice but not in immunodeficient mice (61). Further investigation revealed that lenvatinib modulates antitumor immune responses by reducing tumor-associated macrophages and increasing activated CD8^+^ T cells secreting interferon-γ and granzyme B (62). Lenvatinib prevents Treg differentiation and infiltration, and reverses T cell suppression, by reducing tumor PD-L1 level (106, 107).

As second-line TKIs, regorafenib and cabozantinib also exhibit immunomodulatory effects and antiangiogenetic functions. Regorafenib promoted T cell activation, M1 macrophage polarization, and proliferation/activation of cocultured T cells *via* p38 kinase/Creb1/Klf4 axis, therefore enhancing antitumor immunity independently from its antiangiogenic effects (108). The antitumor effect of T cells is a human leukocyte antigen class I (HLA-I)-dependent immune response. HLA-I is essential for tumor antigen presentation and subsequent antitumor immunity. Tumor cells evade immune detection by acquiring deficiencies in HLA antigen processing and presentation pathways (109). It has been shown that regorafenib can increase the expression of cell surface HLA-I, and upregulate various genes associated with the HLA-I antigen processing pathway, as well as its transcriptional regulators (110). Cabozantinib showed its immunomodulatory activity making murine colon tumor cells more sensitive to immune-mediated killing by altering the phenotype of tumor cells, the proportions of immune cell subpopulations in the peripheral circulation, and the tumor microenvironment (71). Subsequent research revealed that cabozantinib significantly increased the infiltration of neutrophils and reduced the proportions of intratumor CD8^+^PD1^+^ T cells in HCC (111).

## 5 Prospects and challenges

With the progress of molecular biological research, the complex and diverse phenotypes, mechanisms, and therapeutic responses of cancer have been gradually uncovered, which are translated to the clinical treatment of cancer. Reprogramming of cellular metabolism replaces the metabolic processes that operate in most normal tissue cells, maintaining their rapid proliferation, invasion, migration, and metastasis (112). In HCC, for instance, abnormal lipid metabolism triggered by SPIN1/SREBP1/FASN axis has been reported to enhance tumor growth (113). Recently, TKIs have been reported to have effects on the metabolic balance of multiple endogenous metabolic pathways, including lipid metabolism (114). More research on the functioning mechanism of TKIs in tumor treatment is needed to guide the clinical application of TKIs in cancer treatment.

Early-stage clinical studies combining targeted therapy with immunotherapy such as ICIs are ongoing and have shown a synergistic effect, suggesting combined treatment for HCC in the future (115, 116). Investigations have proved the immunoregulatory effects of targeted therapy on tumor microenvironment and immune responses (117), making it a potential sensitizer for adoptive T cell therapy. However, the regulatory mechanism of targeted therapy on immune responses, TME, and adoptive T cells have not yet been elucidated. There is also a need for more direct evidence to confirm the clinical benefits of the combination of targeted therapy and adoptive T cell therapy. Further optimization for the combination therapy is still needed for the selection of targeted therapy and the corresponding immune cells to be affected. Therefore, a comprehensive understanding of combination therapy is necessary to optimize the management of HCC and to bring survival benefits for the patients.

It remains a challenge to assess the effects of combination therapies. For HCC patients treated with targeted therapy or immune therapy, mRECIST and immune RECIST (iRECIST) can be applied (118, 119). Tumor antigens, for example AFP and GPC-3, can provide reference value for efficacy evaluation (13, 120). However, current evaluation methods all showed inevitable low sensitivity and hysteresis. Liquid biopsy can dynamically monitor and reflect the treatment efficacy and can be used as a supplement to imaging, but standardization of the evaluation criteria is still needed (121, 122). ncRNA has been trialed as a novel biomarker in clinical diagnosis and efficacy monitoring, but still needs to be extensively validated (123). The development of specific and sensitive molecular markers for the monitoring of the cancer prognosis and treatment efficacy of HCC is still an urgent issue to be settled.

## Author contributions

LJL, PZ, YC, and LL devised the study, wrote and revised the manuscript. PZ, XW, SH, YWC, YC, LL, and LJL contributed to the literature search and gave insightful suggestions in revising this work. All authors contributed to the article and approved the submitted version.
